# Atypical Chest Pain: An Unusual Presentation of Spinal Metastasis due to Penile Carcinoma

**DOI:** 10.1155/2016/7284070

**Published:** 2016-06-26

**Authors:** Sarah Pywell, Shumaila Hasan, Mohammad Zain Sohail, Georgios Mamarelis, Cameron Dott, Mohammad Taimur Khan, Naveethan Sivanadarajah

**Affiliations:** ^1^Trauma and Orthopaedics Department, Princess Alexandra Hospital, Hamstel Road, Harlow CM20 1QX, UK; ^2^Neurosurgery Department, Royal London Hospital, Whitechapel Road, London E11BB, UK; ^3^Manchester Royal Infirmary, Oxford Road, Manchester M13 9WL, UK

## Abstract

Spinal metastases may present in a myriad of ways, most commonly back pain with or without neurology. We report an unusual presentation of isolated atypical chest pain preceding metastatic cord compression, secondary to penile carcinoma. Spinal metastasis from penile carcinoma is rare with few cases reported. This unusual presentation highlights the need for a heightened level of clinical suspicion for spinal metastases as a possible cause for chest pain in any patients with a history of carcinoma. The case is discussed with reference to the literature.

## 1. Case Report

A 57-year-old gentleman presented to the emergency department with complaints of gradual onset of chest pain for 4 weeks, exacerbated by lying down. Examination was essentially unremarkable. Haematological and biochemical investigations were normal, including normal cardiac enzyme levels, plain chest radiograph, and 12-lead ECG. The patient was diagnosed with gastroesophageal reflux disease and discharged with antacids.

**Figure 1:**
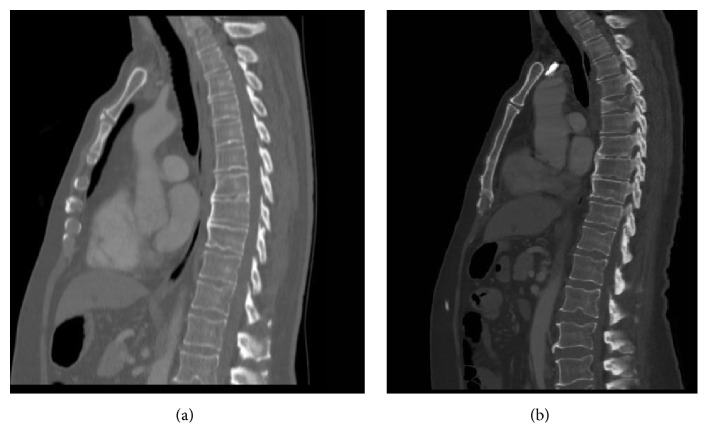
(a) CT scan at the time of penile resection. (b) CT scan at the time of A+E presentation with weakness.

Of note, the patient had a background history of well differentiated squamous cell carcinoma of the penis, for which he had undergone partial penectomy and left sided inguinal node resection 10 months previously, followed by radiotherapy 1 month prior to presentation. No further metastatic disease was found on staging CT at the time of penile resection (Figure 1(a)).

He re-presented one month later with worsening chest pain. Examination revealed vague tenderness around the 4th and 5th intercostal region on the left side. ECG was normal. He was diagnosed with costochondritis and discharged with analgesia. He returned to the emergency department on the following day after a fall secondary to sudden onset bilateral lower limb weakness. He recalled no weakness prior to this or back pain.

Neurological examination revealed increased tone in both lower limbs, globally reduced power bilaterally (MRC class 3), presence of clonus, brisk reflexes, and positive Babinski's reflex, with a vague sensory level just below the costal margin, beyond which sensations were present distally but remarkably reduced in all dermatomes. Rectal examination revealed loss of perianal sensation and anal tone. Bladder scan revealed urinary retention of 900 mL.

Plain thoracic spine radiographs showed partial collapse of T5 vertebra with pedicle destruction which was further evaluated by CT scan (Figure 1(b)).

**Figure 2:**
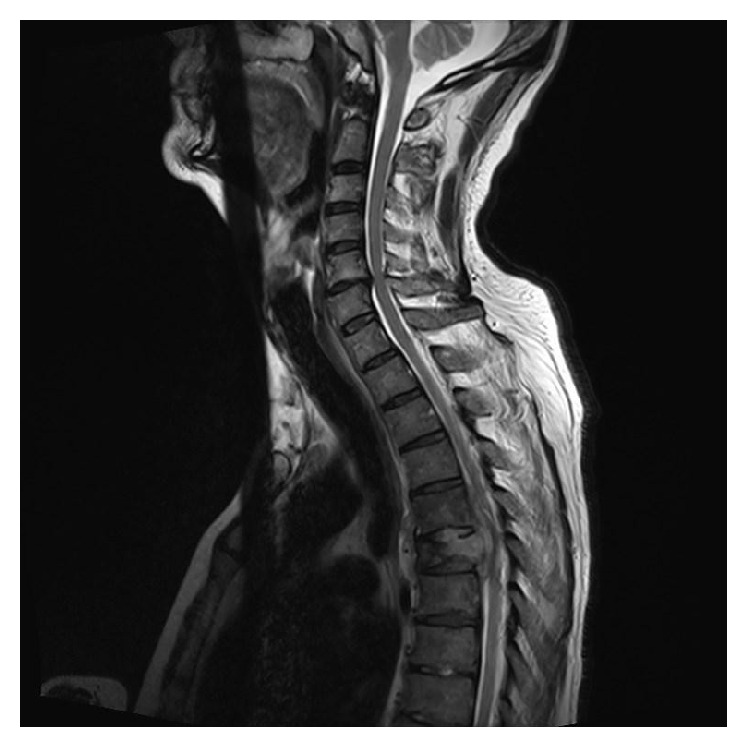
MRI scan, sagittal T2 weighted image, showing posterior soft tissue mass at the level of T5 with associated pathological fracture and diffuse paravertebral oedema. There is evidence of moderate compression of the spinal cord.

**Figure 3:**
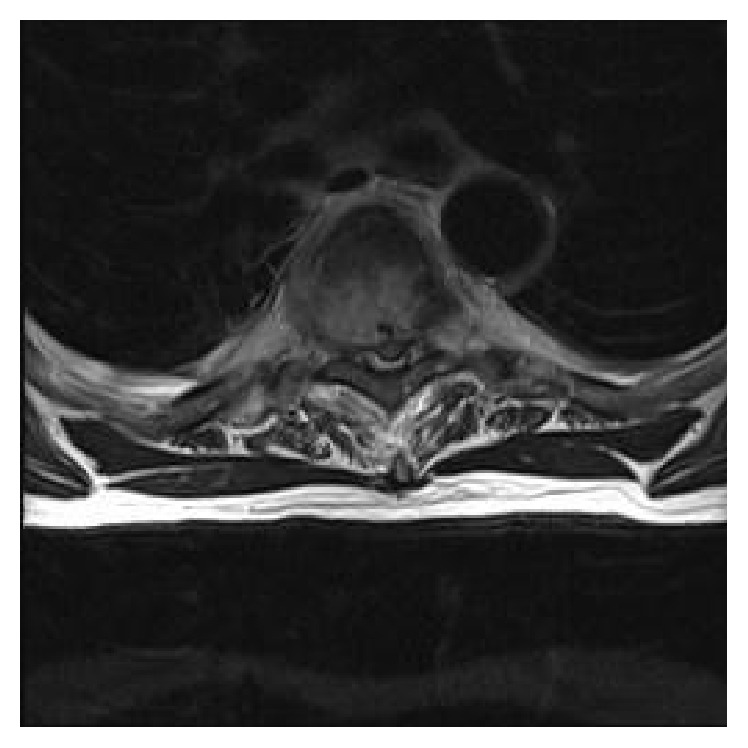
MRI scan, axial T1 weighted image, showing posterior soft tissue mass at the level of T5 with associated pathological fracture and diffuse paravertebral oedema. There is evidence of moderate compression of the spinal cord.

MRI revealed a posterior soft tissue mass at the level of T5 with associated pathological fracture and diffuse paravertebral oedema. There was evidence of moderate compression of the spinal cord. Further small deposits were identified at T2 and T3 vertebrae as well as the right eighth rib (Figures 2 and 3).

Staging CT scan of the chest, abdomen and pelvis revealed multiple retroperitoneal enlarged lymph nodes and focal hepatic lesions.

He underwent urgent spinal decompression and fusion at a regional neurological centre. The histopathology report from the perioperative tissue samples yielded metastatic squamous cell carcinoma. The patient is currently undergoing neurorehabilitation and further oncology follow-up.

## 2. Discussion

Primary penile cancer has an incidence of 1–10% with varying geographical distribution [1] and usually presents in the sixth decade of life. The disease can manifest itself as a locoregional advanced disease in one-third of patients or metastatic disease at presentation in 1-2% of cases [2–4]. Haematogenous spread is rare, ranging from 1 to 3% [5], the commonest sites being the liver and lungs, whilst spread to bone, brain, and skin is less common [5–8].

Penile cancer metastasising to the spine is rare, with only a few cases reported [9–11]. The most common presenting symptom of metastatic disease of the spine from any primary carcinoma is pain, present in 83–95% of patients [12]. In some cases, back pain can precede other symptoms by one year [12, 13]. All previous reports of spinal metastases secondary to penile cancer have presented primarily with back pain [9–11].

The initial presentation in this case was isolated chest pain as opposed to back pain. Chest pain as a presenting symptom of spinal pathology has been previously reported, however with back pain as a concomitant symptom [14–16]. We believe this to be the first case of isolated chest pain in the presence of spinal metastasis to be reported. The likely cause of chest pain may be ascribed to local infiltration compressing the affected thoracic nerve root.

Due to the rarity of spinal metastases from penile carcinoma, no definitive guidelines for treatment exist. In the three previously reported cases of spinal metastasis secondary to penile cancer, two were treated with palliative radiotherapy [9, 10] and one was treated with surgical resection and stabilisation [11] due to rapid progression in neurology emergency decompression was deemed most appropriate to reduce permanent and future neurological deficit.

## 3. Conclusion

Spinal metastasis from penile carcinoma is a rare entity, with no current treatment guidelines, and should therefore be managed in a similar fashion to that from any other primary carcinoma.

The unusual presentation in this case highlights the need for a raised level of clinical suspicion for spinal metastases as a possible cause for chest pain in any patients with a history of carcinoma.
